# Age, Anger, and Gratitude: An Online Emotion Induction to Assess Advice-Taking in Older Age

**DOI:** 10.1177/01640275251362251

**Published:** 2025-07-19

**Authors:** Tarren Leon, Gabrielle Weidemann, Phoebe E. Bailey

**Affiliations:** 1 Graduate School of Health, 1994 University of Technology Sydney, Sydney, NSW, Australia; 2 School of Psychology, 6489 Western Sydney University, Penrith, Australia; 3 MARCS Institute for Brain, Behaviour, and Development, 6489 Western Sydney University, Penrith, Australia

**Keywords:** advice-taking, decision-making, older adults, autobiographical recall, judge-advisor task

## Abstract

Older adults prioritize emotion regulation over other cognitively demanding tasks. Thus, emotions requiring regulation may increase reliance on advice when making judgements. An online sample of 42 young, 48 middle-aged, and 42 older adults were randomly allocated to either an anger, gratitude, or neutral emotion induction, using autobiographical recall. A judge-advisor task measured advice-taking, and participants rated their confidence, perceived advice accuracy, and emotions, followed by the general decision-making styles questionnaire. Due to emotion induction failure, a global positive mood score was explored. Although positive mood did not correlate with advice-taking, greater age was associated with lesser avoidant decision-making style, lower pre- and post-advice confidence, and greater positive mood. Perceived advice accuracy was positively correlated with both pre- and post-advice confidence ratings, positive mood, and advice-taking. The present study provides no evidence for age-related differences in the degree of advice-taking, but suggests that different mechanisms likely underpin advice-taking at different ages.

## Introduction

During decision-making, we often encounter advice, whether it is sought out or not. The extent to which we use this advice can be influenced by our concurrent emotions (de Hooge et al., 2014). These emotions can even be unrelated to the decision at hand (de Hooge et al., 2014; Loewenstein & Lerner, 2009). Older adulthood is a time in which we may experience not only physical changes, but also changes in cognitive, motivational, and emotional processes which can then in turn influence decision-making processes (Bruine de Bruin et al., 2020; Horn & Freund, 2022; Livingstone & Isaacowitz, 2021; Mata et al., 2007). Such changes may also influence how receptive older adults are to advice, which can have health, political, financial, and social implications. With populations of older adults increasing around the world (Van Den Bruele et al., 2019), it is important to investigate how age and emotion processing might interact to influence advice-taking.

### Incidental Pre-Decisional Affect and Advice-Taking

Pre-decisional incidental affect, that is emotions elicited by a source unrelated to a specific decision (de Hooge et al., 2014; Loewenstein & Lerner, 2009), can influence decision-making processes (Raghunathan & Pham, 1999). Even minimal sensory cues have been suggested to contribute to incidental emotions (Loewenstein & Lerner, 2009). Positive emotions have been associated with greater risk-taking decisions in both young and older adults (Chou et al., 2007). A possible explanation for this is that positive mood indicates an absence of threat, leading to greater risk-taking (Magnan & Hinsz, 2005). On the other hand, positive emotions have been proposed to broaden attentional focus (Loewenstein & Lerner, 2009), and have been associated with older adults’ improved performance on a complex decision-making task (Carpenter et al., 2013). In a study investigating negative emotions, Raghunathan and Pham (1999) induced young adults to feel either sadness or anxiety, and found that sadness was associated with more high risk/high reward decisions relative to anxiety. For those feeling anxiety, the reverse was found (i.e., there was a preference for low risk/low reward decisions). This suggests that emotions of the same negative valence can influence decision-making in different ways. Taken together, these studies support the idea proposed by Loewenstein and Lerner (2009), that incidental emotions can have both beneficial or harmful influences on decision-making processes.

Incidental affect can also influence how receptive individuals are to advice (de Hooge et al., 2014). For instance, gratitude, an emotion generally elicited by other people (i.e., an other-focused emotion), has been shown to increase in advice-taking, whereas, pride, an emotion generally elicited by the self (i.e., a self-focused emotion), has been shown to decrease advice-taking (de Hooge et al., 2014). Since both are positive valence emotions, it was determined that the causality of emotions, whether they are elicited by the self or by others, plays a role in how the emotion influences advice-taking. To further illustrate this, they found that when anger was derived from the self, advice-taking increased, whereas anger caused by others decreased advice-taking. Given that older adults tend to show preferences for positive material over negative (Reed & Carstensen, 2012; Reed et al., 2014), and are more adept at regulating their emotions (Orgeta, 2009; You et al., 2019), it is possible that age-related differences may exist in the association between incidental affect advice-taking.

### Older Adults, the Positivity Effect, and Advice-Taking

The positivity effect is an age-related cognitive processing trend in which there develops a preference for positive material relative to negative material (Reed & Carstensen, 2012). Preference towards positive material has been demonstrated in older adults’ attention and memory (see Reed et al., 2014), and appears to also extend to decision-making (Löckenhoff & Carstensen, 2007). The positivity effect is theorised to occur due to motivational processes, as per socioemotional selectivity theory (Carstensen, 2006), or by neural atrophy as proposed by the aging brain model (Cacioppo et al., 2011). In the case of the prior theory, older adulthood is a time in which individuals become more motivated towards emotional goals and wellbeing within the present moment (Reed & Carstensen, 2012). Emotion regulation abilities have thus been proposed to contribute to the effect (Charles & Carstensen, 2013).

Older adults appear to prioritize emotion regulation over other cognitively demanding tasks (Mienaltowski & Blanchard-Fields, 2005; Phillips et al., 2002). While this prioritization can be beneficial for wellbeing in the present moment, the positivity effect may lead to older adults relying more heavily on affective information when making decisions (Peters et al., 2007). Reliance on affective information may then lead to older adults using more automatic decision-making processes, relative to processes that are more deliberative (Peters et al., 2007). According to dual process theories of decision-making, automatic processes are fast, implicit, associative, and intuitive, whereas deliberative processes are slower, more conscious, analytical and reason based (Peters et al., 2007). Additionally, older adults have been reported to be more avoidant in their decision-making (Nolte & Löckenhoff, 2025; Peters et al., 2007), and to prefer deferring decisions to others such as physicians or family members (Chen et al., 2011; Finucane et al., 2002; Pinquart & Duberstein, 2004). In contrast, older adults have recently been shown to consult others less when making decisions (Wild & Löckenhoff, 2024). Nevertheless, the age-related trend towards emotional wellbeing in the present may help to explain an age-related bias towards relying on automatic decision processes, including decision-making avoidance, and potentially other aspects of decision-making such as advice-taking.

It has been suggested that advice can reduce the complexity of a decision (Bonaccio & Dalal, 2006). As such, older adults may be more inclined to rely on advice during decision-making that involves simultaneous and cognitively demanding emotion regulation. Advice may provide a way for older adults to make a quick decision, without having to expend as much cognitive effort. The first study to our knowledge to examine age-related differences in advice-taking found that older adults place more weight on advice than young adults (Bailey et al., 2021). Relative to young adults, older adults also discriminated less between expert and novice sources of advice, and were particularly influenced by novice advice. Similarly, a further study showed that older adults take as much advice as young adults despite perceiving that advice to be less accurate (Leon et al., 2024). As mentioned above, it may be that advice, no matter the source, offers older adults an easier alternative than expending cognitive effort to engage in deliberative decision-making processes in order to simultaneously assess advice and a decision. Indeed, older adults’ higher ratings of the value of novice and expert advice, correlated with poorer working memory and greater fluid intelligence, respectively (Bailey et al., 2021). These findings suggest that age influences advice-taking. However, the potential impact of emotions on older adults’ advice-taking remains to be examined.

### Self-Confidence

It has been proposed that high self-confidence may be a reliable indicator of egocentric discounting in advice-taking (Bonaccio & Dalal, 2006; Morin et al., 2021) – that is, the tendency for individuals to weigh their own estimates more heavily than that of others. In studies investigating confidence and advice-taking among young adults, initial low self-confidence has been found to be a reliable predictor of advice-seeking behaviour (Pescetelli et al., 2021), and for individuals experiencing high levels of confidence, a decrease in advice-taking (i.e., egocentric discounting) has been reported (See et al., 2011). Counter to this latter finding, Pescetelli et al. (2021) showed that the estimates of those who had higher initial self-confidence were more influenced by subsequent advice. The authors reasoned that this counter-intuitive finding may be due to a misattribution of one’s own confidence onto the advice.

Older adults perceive themselves to be worse decision-makers than younger adults (Bruine de Bruin et al., 2012), or find decision-making more challenging (Peterson & Cheng, 2022). However, evidence to date is mixed as to whether, relative to young adults, older adults are overconfident (Burns et al., 2016; Cauvin et al., 2019; Hansson et al., 2008), or less overconfident in their abilities to perform certain cognitive tasks (Bruine de Bruin et al., 2012; Pliske & Mutter, 1996; Smith et al., 2023; Strough et al., 2016). Additionally, confidence can be influenced by mood. Mienaltowski and Blanchard-Fields (2005) found that relative to young adults, older adults induced to experience a positive mood showed lower levels of confidence than those induced to feel a negative mood, and the reverse pattern was found for young adults. Given the varied findings, the current study was designed to explore how confidence, age and mood interact to influence advice-taking.

## Cognitive Declines with Age

The research to date suggests that older adults exhibit a decline in some cognitive abilities, and this appears to relate to age-related differences in decision-making, and advice-taking (Deary et al., 2009; Fechner et al., 2019). Combined with the age-related positivity effect, cognitive declines may result in older adults being more likely to rely on advice in decision-making. Anger in particular is an emotion that can be of a higher intensity, and may require a greater allocation of cognitive resources to regulate (Blanchard-Fields, 2007).

If older adults prioritize regulating emotions over other tasks, and an emotion such as anger requires greater cognitive resources to regulate, it is possible that older adults’ performance on other concurrent tasks will decline if they are experiencing anger. That is, older adults may attend less to a decision and more to mood repair and simply rely more on advice as an easy option to reduce cognitive load. This may contrast with the effects of anger on young adults’ advice taking, where incidental anger correlates with young adults being less receptive to advice (de Hooge et al., 2014). In comparison, when induced to feel a positive emotion such as gratitude, older adults may not need to engage in emotion regulation to repair mood, and thus, may have greater cognitive resources available for decision-making and may therefore rely less on advice. Conversely, recall that incidental gratitude coincides with young adults being more receptive to advice (de Hooge et al., 2014).

### The Current Study

The goal of the present study was to investigate how negative and positive emotions induced in an online environment may influence advice-taking in older age. Drawing upon work by de Hooge et al. (2014), we examined other-focused anger, and other-focused gratitude – emotions said to decrease and increase young adults’ advice-taking, respectively. It was hypothesised that, in an adult lifespan sample, older age would be associated with greater reliance on advice. Relative to a control condition with no emotion induction, a decrease in advice-taking was anticipated for those induced to feel anger. This decrease was expected to be smaller as age increased. Relative to the control condition, gratitude was expected to increase advice-taking averaged across age. In addition, we sought to explore the effects of global positive mood, confidence, decision-style, and perceived accuracy of advice on advice-taking.

## Methods

### Participants

Forty-four young, 49 middle-aged, and 43 older adults participated in the current study. An a priori power analysis using G*Power (Faul et al., 2007) showed that to detect a medium sized effect (*f* = .15) of age on advice-taking (Bailey et al., 2021) for a multiple linear regression with four predictors, α = .05 and 95% power, 129 participants (i.e., 43 young, 43 middle-aged, 43 older) were required. All participants were recruited online via *Qualtrics Panels* for a fee of $15 per participant. All participants resided in Australia. Data for four participants (young group: 2 males; middle-aged group: 1 female; older group: 1 male) were removed from analyses due to reporting a current neurological condition. The final sample consisted of 42 young (*M* age = 27.50 years, *SD* = 5.83; range = 19–39; 22 female), 48 middle-aged (*M* = 50.40 years, *SD* = 7.51; range = 40–64; 24 female), and 42 older adults (*M* = 71.00 years, *SD* = 5.93; range = 65–89; 21 female). As shown in Table 1, the age groups did not significantly differ on ratings of health or years of education. All participants gave written informed consent, and the research was approved by the Western Sydney University Human Ethics Committee, approval number (H12559). The study was pre-registered via *AsPredicted* (#52839) https://aspredicted.org/IJL_XOP.

**Table 1.** Descriptive Statistics and Age Group Differences for Health and Education Measures.

	Young adults		Middle-aged adults		Older adults		Inferential statistics			
Variable	*M*	*SD*	*M*	*SD*	*M*	*SD*	*F*	*p*	*df*	*η*_*p*_^2^
Education	15.00	3.14	14.60	4.97	13.80	3.28	1.11	.333	129	.02
Health	5.10	1.38	4.96	1.38	4.62	1.61	1.20	.305	129	.02

*Note.* Health is rated on a scale from 1 (poor) to 7 (excellent).

### Materials and Procedure

#### Judge-Advisor Task

The judge-advisor task provides a measure of advice-taking (Sniezek & Buckley, 1995). Typically, participants provide initial quantitative estimates on a given topic, receive numeric advice about the topic, and then provide revised estimates. We asked participants to estimate the number of coins in a jar. This was to ensure the age groups were equated on base-level knowledge of the task at hand. We did not expect any of the age groups to be more or less knowledgeable in identifying the numbers of coins in a jar.

Prior to the main task, each participant completed three practice trials without advice. This practice phase was included to allow participants to become familiar with the task. It also allowed for calculation of opinion difference scores (i.e., initial estimate – actual number of coins in the jar) to check for any age group differences in base level knowledge. Following the three practice trials without advice, participants were shown the same images, one at a time, along with their first estimate and advice. The advice was within +/− 5% of the actual value. After entering a second estimate, participants were presented with the correct value of coins in the jar to facilitate learning about the task. These images of jars filled with coins differed from those used in the main task.

The first round of the main judge-advisor task involved 12 trials, each with an image of a jar filled with coins. The number of coins in each jar differed, and the order of image presentation was randomised for each participant. Following the first round of the task, participants completed an emotion induction procedure, as described below. For the second round of the task, participants saw the same twelve images, one at a time, along with their own initial estimates and the advice. The advice was not framed as coming from anyone in particular, nor were participants provided any feedback or detail about the advice accuracy. However, the actual advice was always within +/− 5% of the correct number. Reliance on advice is measured using the *weight of advice* calculation: [(final estimate – initial estimate) / (advice – initial estimate)]. A weight of advice score of zero indicates no reliance on advice, while a score of 1 indicates complete reliance on advice.

#### Stimuli

The first author created the stimuli for this experiment using household mason jars and Australian coins. One jar was used for all images in the main task, and a different jar was used for the images in the practice phase. Differing amounts of coins were used for each image. The number of coins in the jars ranged from 36 to 273.

#### Emotion Induction

Following previous studies (de Hooge et al., 2014), an autobiographical recall task was used to induce other-focused gratitude, and other-focused anger. Participants were instructed to vividly recall a situation in which they felt the target emotion. They were told to think about this situation in as much detail as possible, and then answer the following questions: “Who was the person you felt gratitude (*anger*) towards?”; “What is your relation to this person?”; “Where were you when you felt gratitude (*anger*) towards this person?”; “Why did you feel gratitude (*anger*) towards this person?”; “What impact did this situation have on you?”; “Is there anything else that stands out from this experience?”. Participants were instructed to include as much detail as possible in their written responses, and to write in complete sentences. Control group participants were asked to think of a normal weekday, and were asked questions relating to this: that is “Who is usually involved in your normal weekday?”; “Where does a normal weekday usually take place for you?”; “What do you usually do in your normal weekday?”; “Are there any other tasks that you do during a normal weekday?”.

As a manipulation check for this induction, participants rated the extent to which they were feeling a list of emotions in that current moment (fear, joy, anger, surprise, disgust, gratitude, sadness) on a scale from 1 (not at all) to 5 (very much). These emotion ratings were collected immediately prior to the emotion induction (after the first round of the judge-advisor task), and again after the second round of the judge-advisor task.

#### Global Positive Mood

Each participant’s average pre-induction and post-induction negative emotion ratings (anger, disgust, sadness) were subtracted from pre-induction and post-induction positive ratings (joy, gratitude) to calculate a global positive mood score.

#### Rated Decision-Making Confidence

At the end of each judge-advisor round (i.e., pre- and post-advice), participants rated how confident they were in their own estimates on a scale from 1 (not at all confident) to 5 (very confident).

#### Rated Advice Accuracy

Following the end of the second round of the judge-advisor task, participants rated how accurate they thought the advice was on a scale of 1 (not at all) to 5 (very).

#### General Decision-Making Styles Questionnaire (GDMS)

The GDMS (Scott & Bruce, 1995) questionnaire provides a measure of the extent to which individuals use rational (e.g., “My decision-making requires careful thought”), intuitive (e.g., “I generally make decisions that feel right for me”), dependent (e.g., “I rarely make decisions without consulting other people”), avoidant (e.g., “I postpone decision-making whenever possible”), and spontaneous (e.g., “I make quick decisions”) decision-making styles in their everyday lives. The questionnaire involves 25 items rated on a 5-point response scale from 1 (strongly disagree) to 5 (strongly agree). The word “important” was removed from statements relating to the dependent style, in order to keep the questionnaire relative to day-to-day decision-making. Cronbach’s alphas for the present study were .89 for Rational, .82 for Intuitive, .82 for Dependent, .90 for Avoidant, and .83 for Spontaneous.

### Procedure

The study was conducted online using Qualtrics. Participants first provided informed consent. They then completed the background information questionnaire, followed by the practice trials of the judge-advisor task. Round 1 of the judge-advisor task (estimates without advice) followed this. At the end of round 1, they provided confidence and emotions ratings. Participants were then randomly allocated to an emotion induction (or control) condition, completing the induction procedures described above. Round 2 of the judge-advisor task was then completed (estimates with advice), as were ratings of confidence, advice accuracy, and emotions ratings. Finally, all participants completed the GDMS questionnaire, and were then debriefed.

#### Data Preparation

Following Bailey et al. (2021) and Wang and Du (2018) as well as our preregistered plan, trials were removed where the initial estimate matched the advice (young: .08% of practice phase trials only, middle-aged: .07% of practice phase trials and .02% trials proper, older: .08% practice phase trials and .02% trials proper), thus preventing a calculation of advice-taking. In addition, weight of advice values greater than 1.3 or less than −1.3 were removed. Values greater than 1.3 would incorrectly indicate someone being highly influenced by the advice, when they have actually moved further away from it. This resulted in the removal of 7% of trials from the older group, 6% of trials from the middle-aged group, and 5% from the young group.

#### Outlier Data

Following our preregistered plan, scores more than +/− 3 *SD* from the age group mean were identified as outliers and adjusted to the mean +/− 3 *SD*. Among the young group, this resulted in adjustment of one GDMS score (rational style), and one weight of advice value. Among the middle-aged group, three emotions ratings, one GDMS score (rational style), and five weight of advice values were adjusted. Among the older group, six emotions ratings, and four weight of advice values were adjusted. All analyses were conducted in *R-Studio* (RStudio Team, 2020) version 4.2.1.

## Results

### Judge-Advisor Practice Phase

Using the rstatix (Kassambara, 2021) package, an Age Group (Young, Middle-age, Older) × Practice Trial (1, 2, 3) Analysis of Variance (ANOVA) was conducted on opinion difference scores. A main effect of Practice Trial, *F* (2, 247) = 8.98, *p* < .001, *η*_*p*_^2^ = .07, indicated that across the age groups, the opinion difference became smaller from the first practice trial to the second practice trial (*p* < .011), and remained similar between the second practice trial and third practice trial (*p* = .681). This indicates rapid learning by the participants. There was no main effect of Age Group, *F* (2,129) = 2.94, *p* = .057, *η*_*p*_^2^ = .04, and no Age Group × Practice Trial interaction, *F* (4, 246) = 0.92, *p* = .452, *η*_*p*_^2^ < .01. Average opinion differences by each age group are presented in Supplemental Table S1, and a graph of the average opinion differences can be seen in Supplemental Figure S1.

### Mixed Effects Models

Where mixed effects models were used in the following analyses, continuous fixed effects predictors were grand-mean centered, as this is suitable for analyses of between-subjects effects (Enders & Tofighi, 2007). As such, the baseline of the continuous predictor variables are the average values for that respective predictor variable (i.e., Age β estimates would indicate the change in the outcome variable per 1-unit change from the average age). Model selections were determined by Loglikelihood Ratio Test results, as these can be useful for simple comparisons of nested models (Meteyard & Davies, 2020). Where normality of residuals were violated, as indicated by quantile-quantile (QQ) plots and significant Shapiro Wilk’s tests, parametric model analyses were retained as it has been suggested that violations of normality in Gaussian models are robust to such violations and can still be the preferable over more specified non-parametric approaches, which can be prone to error (Knief & Forstmeier, 2021; Schmidt & Finan, 2018). Analyses were conducted using the lme4 (Bates et al., 2015), sjPlot (Lüdecke, 2021), and lmerTest (Kuznetsova et al., 2017) packages. *p*-values and 95% Confidence Intervals (CIs) were computed using Satterthwaite approximation.

### Emotion Inductions

To assess whether the emotion inductions were successful at eliciting the target emotions, mixed effects models were created for the anger and gratitude ratings respectively. The emotion ratings served as the outcomes, and fixed effects predictors consisted of Time (Pre-Induction, Post-Induction), Condition (Control, Anger, and Gratitude groups), Age (as a continuous variable), and interactions. Participant ID was set as a random effect. Of particular interest were the effects of Time and Condition on the emotion ratings—i.e., the Anger and Gratitude ratings as a function of the respective emotion induction, from pre-induction to post- induction. As such, models with these two predictors served as a baseline for further comparison and fit. For the categorical variable of Time, Pre-induction served as the reference level, and for the categorical variable of Condition, the Control served as the reference level. We expected significant positive effects for the level of Time at Post-Induction, and for Condition at the level of Anger (for the anger ratings), and Gratitude (for the gratitude ratings).

#### Anger Ratings

Our findings indicated that the emotion induction failed to elicit anger—i.e., there were no positive effects of Time (post-induction), or Condition (specifically, Anger), on the anger ratings. Model comparisons can be seen in Supplemental Table S3, and the final model is reported in Supplemental Table S3. Both Time (Post-Induction) (β = −0.12, *SE* = 0.06, 95% CI [−0.24, 0.00], *t* (131) = 1.89, *p* = .061), and Condition (Anger) (β = −0.32, *SE* = 0.18, 95% CI [−0.67, 0.34], *t* (128) = 1.75, *p* = .082) had negative effects on anger ratings. Interestingly, there was a significant effect of Age in a negative direction (β = −0.02, *SE* = 0.00, 95% CI [−0.03, −0.01], *t* (128) = 4.84, *p* < .001).

#### Gratitude Ratings

Model comparisons can be seen in Supplemental Table S4, with the final model reported in Supplemental Table S5. Again, our findings indicated that the emotion induction failed to elicit the target emotion. Both Time (Post-Induction) (β = −0.08, *SE* = 0.08, 95% CI [−0.23, 0.08], *t* (131) = 0.97, *p* = .333), and Condition (Gratitude) (β = −0.37, *SE* = 0.23, 95% CI [−0.83, 0.08], *t* (129) = 1.60, *p* = .112) had negative effects on gratitude ratings.

### Correlations with Weight of Advice

Using the Hmisc (Harrell, 2022) and psych (Revelle, 2023) packages, correlation matrices using Spearman’s correlation (due to the ordinal scale of data) analysed the relationships between age, the five decision-making styles, the average weight of advice, pre- and post-advice confidence, perceived advice accuracy ratings, and global positive mood. Education was included as a covariate.

Results are displayed in Table 2. Age had a negative correlation with pre-advice, and post-advice, confidence ratings. There was also a negative relationship between age and the avoidant decision-making style. Education had a positive correlation with the rational decision-making style, and the post-advice confidence ratings. There was also a negative correlation between education and the avoidant decision-making style. The rational decision-making style had a positive correlation with the post-advice confidence, as well as the perceived advice accuracy. The dependent decision-making style had a positive correlation with perceived advice accuracy. Pre-confidence ratings were positively correlated with the post-confidence ratings, and both pre-confidence and post-confidence were positively related to perceived advice accuracy. Global mood positively correlated with age, pre-advice confidence ratings, post-advice confidence ratings, and perceived advice accuracy. There were also positive correlations between global mood and the rational and intuitive decision-making styles. Perceived advice accuracy had a positive correlation with the average weight of advice.

**Table 2.** Intercorrelations Between Age, Decision-Making Styles, Confidence Ratings, Perceived Advice Accuracy, Global Positive Mood, and Average Weight of Advice Among the Participants.

Variable	1	2	3	4	5	6	7	8	9	10	11
*n*	132	132	132	132	132	132	132	132	132	132	132
1. Age											
2. Education	−0.13										
3. Rational style	0.11	0.20*									
4. Intuitive style	0.15	−0.05	0.39***								
5. Dependent style	−0.10	−0.06	0.07	0.13							
6. Avoidant style	−0.26**	−0.23*	−0.22*	−0.07	0.43***						
7. Spontaneous style	−0.15	−0.07	−0.22*	0.28**	0.20*	0.33***					
8. Pre-advice confidence	−0.30***	0.06	0.11	0.10	0.14	0.15	0.13				
9. Post-advice confidence	−0.25**	0.21*	0.20*	0.13	0.15	0.12	0.07	0.65***			
10. Perceived advice accuracy	−0.13	0.05	0.19*	0.03	0.24*	0.15	−0.04	0.31***	0.45***		
11. Global positive mood	0.18*	−0.02	0.19*	0.22*	−0.01	−0.02	−0.03	0.25**	0.29**	0.22*	
12. Average weight of advice	0.08	−0.06	−0.09	−0.01	−0.03	−0.15	−0.04	−0.09	−0.03	0.27**	0.14

*Note.* * indicates *p* < .05. ** indicates *p* < .01. *** indicates *p* < .001.

## Discussion

The current study aimed to investigate how negative and positive emotions may influence advice-taking and decision-making across adulthood. The emotion induction, using an online version of an autobiographical recall task, was intended to facilitate examination of the specific emotions of anger and gratitude. Due to failure of the emotion inductions to elicit the target emotions, we were unable to test our hypotheses explicitly relating to induced anger and gratitude. However, regardless of emotion, and contrary to our predictions, advice-taking did not increase with age. The results of the exploratory analyses indicated that an older age was correlated with higher global positive mood ratings and reduced avoidant decision-making style. Interestingly, an older age was also associated with lower pre- and post-advice confidence ratings.

### Age, Incidental Emotions and Advice-Taking

The autobiographical recall emotion induction procedure used in the present research mimicked autobiographical recall inductions used in previous online emotion and advice-taking studies involving young adults (de Hooge et al., 2014). Emotion induction using autobiographical recall has been successful in lab-based studies involving older adults samples (Larcom & Isaacowitz, 2009; Magai et al., 2006; Malatesta et al., 1987; Malatesta-Magai et al., 1992). Despite this, our online inductions failed to elicit the target emotions of anger and gratitude. The exact reason for this failure is unclear, however, we speculate that it may have been due to the online nature of the experiment. Previous studies were conducted in laboratories that may have minimised distractions. It is therefore possible that participants in the current study failed to attend fully to the requirements of the induction due to potential distractions in their uncontrolled environments. Another possibility is that gratitude and anger cannot be completely separated, especially for older adults who may feel forgiveness and positive emotions at the same time as recalling an event that previously elicited anger.

The emotion manipulation check revealed a negative effect of age on anger ratings. This may be indicative of a lack of anger emotions in general with age (Blanchard-Fields, 2007). One study has reported that older adults interpret anger events with more sadness (Kunzmann et al., 2017). It is therefore possible that sadness is a more applicable negative emotion to investigate when examining age effects. While sadness has not yet been examined in terms of its influence on advice-taking, a recent study showed that age did not influence the effect of depressive symptoms on advice-taking (Leon et al., 2024). Research also indicates that aging is associated with greater preference for low-arousal, relative to high-arousal, emotions (Scheibe et al., 2013). This age-related preference may also explain the negative association between age and anger (a high-arousal emotion) in the current study. Future research should consider the arousal states of particular emotions and take into account possible age-related difference in preferences.

In a recent study, age was not associated with advice-taking (Leon et al., 2024). This is consistent with the current data, but not with a previous study demonstrating greater reliance on advice by older adults relative to young adults (Bailey et al., 2021). Inconsistent findings in the literature could potentially be attributed to methodological differences. The present study did not label or provide information about advisors while Bailey et al. referred to advisors as either experts or novices. It is possible that in the context of social decision-making, having some kind of reference to the attributes of the advisor is important in order to detect age-related differences in advice-taking. Judge-advisor tasks that involve knowledge-related versus socioemotional judgments may explain age-related differences in advice-taking. In line with socioemotional selectivity theory, this could potentially be attributable to young versus older adults differing motivations to gain knowledge versus emotional satisfaction, respectively (Carstensen, 2006). Future studies should investigate whether age-related differences in advice-taking exist after manipulating whether advisor attributes are known versus unknown.

Emotion induction failures aside, we found a positive relationship between global positive mood ratings and age. This finding may be underpinned by the positivity effect, and a general predisposition towards more positive mood with age. Our results also indicated an association between greater perceived accuracy of advice and a higher degree of advice-taking. This is in line with a recent meta-analysis showing that perceived advice accuracy was the only unique moderator of advice-taking (Bailey et al., 2023). Social learning models suggest that the attributes of an advisor, such as how knowledgeable, reliable, and confident they are, act as evidence to assist a judge in determining the quality of advice and therefore the extent to which they may rely on advice or learn from the advisor (Hawthorne-Madell & Goodman, 2019). However, the present study did not provide participants with any information about advisor attributes, and it is therefore possible that advice-taking reflected the decision-makers confidence in their own ability.

### Internal States and Advisor Attributes

In the absence of any explicit advisor attribute cues, the participants may have used their own knowledge to infer advisor attributes – something that would reflect a flexible social learner according to Hawthorne-Madell and Goodman (2019). Indeed, there was a positive relationship between the participants’ pre- and post-advice self-confidence ratings and the perceived advice accuracy, as well as a positive relationship between positive mood and perceived advice accuracy. Participants may have used their own feelings of confidence and positive mood to infer the accuracy of advice and thus the knowledgeability and/or confidence of the advisor. This would be in line with the proposition by Pescetelli et al. (2021), that one’s own internal confidence may be attributed to other external factors. Moods have also been suggested to bias cognitive processes (Gross, 2015), thus it is possible that the positive mood participants experienced guided how they processed the available information (i.e., their own internal states), which then led to misattribution or inference of the mood to the environment (Peters et al., 2007). Positive mood may have combined with participants’ confidence to infer greater accuracy of the advice, leading to advice-taking. Of course, it is important to note that only perceived accuracy, and not confidence ratings or positive mood were found to correlate with advice-taking in the present study.

Counter to previous studies suggesting older adults show overconfidence (Burns et al., 2016; Cauvin et al., 2019; Hansson et al., 2008), our results indicated less confidence with age, both before and after receiving advice. This difference may be explained by methodological differences. While the previous studies used tasks that relied more on cognitive performance (Burns et al., 2016; Cauvin et al., 2019; Hansson et al., 2008), the present study was a simpler estimation task. The current results align with a previous study showing that older adults in a positive mood induction condition were less confident than older adults in a negative mood induction when making attributions about another person’s disposition (Mienaltowski & Blanchard-Fields, 2005). Indeed, the current data indicated a positive correlation between age and global positive mood. That is, with older age there may be more positive global mood, which in turn may be related to reduced confidence with age, particularly when inferring the disposition of another person or an advisor.

### Age and Avoidant Decision-Making

While dual process models of decision-making have suggested that older age increases reliance on more automatic decision-making processes (Peters et al., 2007), our decision style data suggest the opposite. The only decision-making style that correlated with age in the present study was the avoidant style. However, this correlation was not in line with our predictions, nor with associations reported in previous work (Chen et al., 2011; Mather, 2006). Our results indicated that with increased age, there is reduced reliance on an avoidant decision style. This finding contrasts with past research and may be explained by the nature of the decisions assessed in previous research, which have largely focused on health (Finucane et al., 2002) and finances (Chen et al., 2011), or by differing cultural backgrounds of participants in each study.

The decision-making style questionnaire used in the present study was aimed at investigating everyday decision-making. Avoidant decision style, as indexed by this measure, was not associated with advice-taking. As suggested by Mather (2006), it may be that older adults are more inclined to avoid decisions when those decisions are stressful. Future research investigating decision avoidance in older age, while manipulating levels of stress, could provide valuable insight into how older adults might adapt their decision style to the specific decision-making context. However, it must be noted that Nolte and Löckenhoff (2025) recently found no evidence for an association between peri-decisional affect (i.e., stress and anticipated regret) and decision avoidance, averaged across age.

## Conclusion

The present study contributes to the very limited evidence base regarding the effect of adult age on the incorporation of advice into decision-making. The data show that older age is associated with more positive mood and less pre- and post-advice confidence in the decision at hand. The only variable to correlate with greater advice-taking was greater perceived advice accuracy, and averaged across age, greater confidence was associated with perceiving advice to be more accurate. However, there was no association between age and advice-taking. A previous meta-analysis indicated no effect of age on advice-taking, but only included one study with adults aged more than 65 years (Bailey et al., 2023). The present study adds to growing evidence that age may not influence advice-taking, but that different mechanisms (e.g., confidence) are likely to underpin advice-taking at different ages.

## Supplemental Material

Supplemental Material - Age, Anger, and Gratitude: An Online Emotion Induction to Assess Advice-Taking in Older Age Supplemental Material for Age, Anger, and Gratitude: An Online Emotion Induction to Assess Advice-Taking in Older Age by Tarren Leon, Gabrielle Weidemann, Phoebe E. Bailey in Research on Aging

## Consent to Participate

Participants provided informed consent.

## Data Availability

The datasets generated and analysed during the current study are available in the OSF repository, https://osf.io/c5xu4/?view_only=1d0bc8bfa3bc4bc9b19f055a6f4e8603.
