# Supplementary material for: Age, Anger, and Gratitude: An Online Emotion Induction to Assess Advice-Taking in Older Age
Source: Res Aging. 2025 Jul 19;48(2):158–68. doi: 10.1177/01640275251362251 (PMC12705867; doi:10.1177/01640275251362251)
Supplement: Supplemental Material - Age, Anger, and Gratitude: An Online Emotion Induction to Assess Advice-Taking in Older Age [file sj-pdf-1-roa-10.1177_01640275251362251.pdf]

Supplementary Materials

Table S1

Average Opinion Difference by Age Group.

Age group			*D*	S
Young adults	26	49.43	3.91	5
Middle-aged adults	44	14.48	05.42	1
Older adults	26	43.81	00.17	1

Figure S1

Opinion difference across the practice trials by age groups. Error bars represent standard errors.

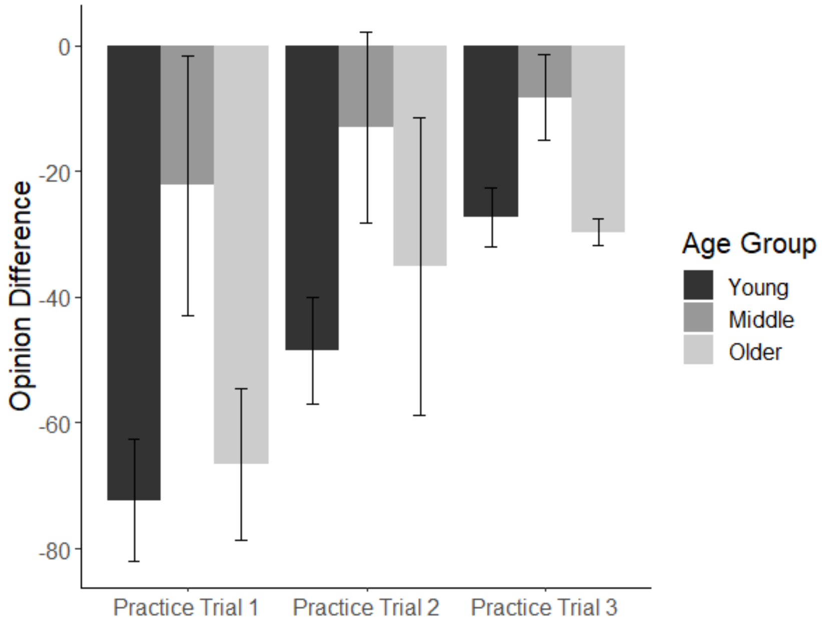

Table S2

Anger Model Comparisons.

Model specification	Model name	Nested / Simpler model	Fixed Effects added	Model fit				LRT Test against nested	
Sampling Units		*N* Total Observations = 264<br>*N* Subjects = 132							
Random Effects		Subjects = Intercepts<br>Items = None							
				AIC	BIC	LL	npar	*df*	χ^2
Condition and time main effects	Main effects 1	-	Condition + Time	633.32	654.78	-310.66	6		
Condition × Time interaction	Interaction 1	Main effects 1	Condition × Time	636.07	664.68	-310.04	8	2	1.25
Age main effects	Main effects 2	Main effects 1	Condition + Time + Age	613.16	638.19	-299.58	7	1	**22.16***
Condition × Age interaction	Interaction 2	Main effects 2	(Condition × Age) + Time	614.23	646.42	-298.12	9	2	2.93
Time × Age interaction	Interaction 3	Main effects 2	Condition + (Time × Age)	613.51	642.12	-298.76	8	1	1.65

Note. AIC = Akaike's Information Criteria, BIC = Bayesian Information Criteria, LL = LogLikelihood, LRT = Likelihood Ratio Test, npar = Number of Parameters.

Table S3

Final Anger Model.

Fixed Effects					
	Estimate/Beta	*SE*	95% CI	*t*	*p*
Intercept	1.82	0.12	1.59, 2.05	15.39	<.001
Condition (Gratitude)	-0.12	0.17	-0.45, 0.20	0.74	.463
Condition (Anger)	-0.32	0.18	-0.67, 0.03	1.75	.082
Time (Post-Induction)	-0.12	0.06	-0.24, 0.00	1.89	.061
Age	-0.02	0.00	-0.03, -0.01	4.84	<.001

Random Effects			
	Variance	*SD*	ICC
Participant (Intercept)	0.55	0.74	0.69

Model Fit		
R^2	Marginal	Conditional
	0.15	0.73

Note. Model equation in R: Anger Ratings ~ Condition + Time + Age + (1|Participant). Beta regression estimates are unstandardized.

Table S4

Gratitude Model Comparisons.

Sampling Units		N Total Observations = 264<br>N Subjects = 132							
Random Effects		Subjects = Intercepts<br>Items = None							
Model specification	Model name	Nested / Simpler model	Fixed Effects added	Model fit				LRT Test against nested	
				AIC	BIC	LL	npar	df	χ^2
Condition and time main effect	Main effects 1	-	Condition + Time	764.96	786.42	-376.48	6		
Condition x Time interaction	Interaction 1	Main effects 1	Condition x Time	768.25	796.86	-376.12	8	2	0.72
Age main effects	Main effects 2	Main effects 1	Condition + Time + Age	766.38	-376.19	-299.58	7	1	0.58

Note. AIC = Akaike's Information Criteria, BIC = Bayesian Information Criteria, LL = LogLikelihood, LRT = Likelihood Ratio Test, npar = Number of Parameters.

Table S5

Final Gratitude Model.

Fixed Effects					
	Estimate/Beta	*SE*	95% CI	*t*	*p*
Intercept	2.99	0.16	2.67, 3.31	18.22	<.001
Time (Post-Induction)	-0.08	0.08	-0.23, 0.08	-0.97	.333
Condition (Gratitude)	-0.37	0.23	-0.83, 0.08	-1.60	.112
Condition (Anger)	-0.37	0.25	-0.86, 0.132	-1.46	.147

Random Effects			
	Variance	*SD*	ICC
Participant (Intercept)	1.12	1.06	0.74

Model Fit		
R^2	Marginal	Conditional
	0.02	0.74

Note. Model equation in R: Gratitude Ratings ~ Condition + Time + (1|Participant). Beta regression estimates are unstandardized.
